# *Saussurea involucrata* Cultures for High-Altitude Illness: Enhancing Hypoxia Tolerance and Protecting Against Acute/Chronic Hypoxic Injury

**DOI:** 10.3390/nu18040556

**Published:** 2026-02-07

**Authors:** Jinyu Zhao, Yutong Li, Fan Wang, Kangjie Jia, Ge Lou, Huihui Shao, Mingji Jin, Zhonggao Gao, Xianfu Wu, Shuangqing Wang

**Affiliations:** 1 State Key Laboratory of Bioactive Substance and Function of Natural Medicines, Institute of Materia Medica, Chinese Academy of Medical Sciences & Peking Union Medical College, Beijing 100050, China; 2 National Institutes for Food and Drug Control, Beijing 102629, China; 3 Shanxi Kangbao Biological Products Co., Ltd., Changzhi 046000, China; 4 Shanxi Kangbao Biotechnology Co., Ltd., Changzhi 046600, China

**Keywords:** *Saussurea involucrata* cultures, hypobaric hypoxia, hypoxia tolerance, metabolic disorders, functional foods

## Abstract

**Objective**: To systematically evaluate the potential of *Saussurea involucrata* cultures (SICs) against high-altitude illness under hypobaric hypoxia and establish a progressive experimental evidence chain covering acute hypoxia tolerance enhancement and acute/chronic hypoxic injury protection. **Methods:** A tiered experimental strategy was employed. Key findings were derived from primary rat models of acute (5500 m, 8 h) and chronic intermittent (5500 m, 8 h/d, 4–8 weeks) hypobaric hypoxia. A mouse acute tolerance model (10,000 m lethality, closed-system endurance) provided supplementary verification. Comprehensive analyses included survival, hemorheology, multi-organ function, and core mechanistic indicators of endothelial function and oxidative stress. Diamox, Rhodiola, and Compound Danshen Dripping Pills served as positive controls. Normoxic/hypoxic blank groups served as negative controls. **Results:** SICs significantly enhanced acute hypoxia tolerance in mice. In the rat models, SICs demonstrated dose-dependent and selective regulation of the endothelial–oxidative stress/hemorheology axis. Specifically, it downregulated endothelin-1, upregulated nitric oxide, enhanced total antioxidant capacity, and improved chronic hypoxia-induced blood hyperviscosity. Medium doses showed consistent optimal efficacy. SICs had limited effects on macroscopic organ remodeling. **Conclusions:** The core protective effect of SICs lies in enhancing hypoxic tolerance and selectively modulating the interconnected pathways of endothelial function, oxidative stress, and microcirculatory health. This mechanistic profile supports its potential as a preventive or early adjuvant intervention for high-altitude illness, providing a systematic preclinical foundation for translational development.

## 1. Introduction

The high-altitude environment is characterized by hypobaric hypoxia, which disrupts systemic oxygen homeostasis and triggers a spectrum of high-altitude illnesses ranging from mild discomfort to life-threatening conditions [1]. Acute symptoms typically manifest as headache, fatigue, and sleep disturbances, while severe cases may culminate in high-altitude pulmonary edema or high-altitude cerebral edema. Prolonged exposure can lead to pulmonary vascular remodeling, hematopoietic abnormalities, and multi-organ functional impairment [2,3,4,5]. Given the increasing prevalence of high-altitude tourism, engineering projects, emergency rescue, and military operations, there is a pressing need for deployable, safe, and easily implementable strategies to mitigate hypoxic injury.

Nevertheless, current strategies for preventing and treating high-altitude illness have significant limitations [6]. Common physical interventions, such as staged acclimatization, oxygen supplementation, and hyperbaric chambers, are often constrained by logistical factors, making rapid deployment in emergency rescue scenarios difficult. Although chemical agents like acetazolamide and glucocorticoids demonstrate clear efficacy, long-term administration frequently leads to adverse effects, including electrolyte imbalances and gastrointestinal disturbances. Furthermore, these drugs are contraindicated in specific populations, such as pregnant women and individuals with hepatic or renal impairment [7,8]. Consequently, natural products have garnered attention as research hotspots due to their multi-target regulatory potential; however, they face two major bottlenecks, the first of which is inconsistent quality. The active ingredient content in herbal medicines such as Rhodiola and *Astragalus membranaceus* varies significantly depending on geographical origin and harvest time, resulting in the poor reproducibility of therapeutic effects [9]. The second limitation is resource sustainability. *Saussurea involucrata*, a wild medicinal plant, has become endangered due to its strict ecological requirements and over-exploitation. Meanwhile, artificial cultivation is hampered by long growth cycles and low yields [10,11].

To overcome the aforementioned bottlenecks, this study utilized plant cell engineering to establish *Saussurea involucrata* cultures (SICs), enabling stable, large-scale production via in vitro cultivation. Concurrently, an HPLC fingerprinting quality control system was developed using syringin as the reference compound [12]. The similarity of ten batches of SICs samples exceeded 0.9, and nine common peaks were identified to ensure batch-to-batch consistency. The traditional medicinal value of snow lotus is closely related to the various active ingredients it contains, including phenylpropanoids (such as syringin), flavonoids, and terpenoids. These components are believed to be associated with its anti-inflammatory, antioxidant, and other pharmacological effects [13]. Syringin, as one of the characteristic phenylpropanoid glycosides, was selected as the chemical reference for this quality control system. This strategy not only circumvents the resource scarcity of wild medicinal plants but also guarantees the chemical stability and traceability of the experimental material, thereby establishing a reproducible foundation for pharmacodynamic studies. Previous studies have demonstrated that *Saussurea involucrate* and its constituents can alleviate hypoxia-induced discomfort via antioxidant and anti-inflammatory mechanisms [10]. However, as a product of a standardized culture system, the specific effects and underlying mechanisms of SICs on the entire spectrum of high-altitude hypoxia—from tolerance and injury to adaptation—remain to be elucidated.

**Figure 1:**
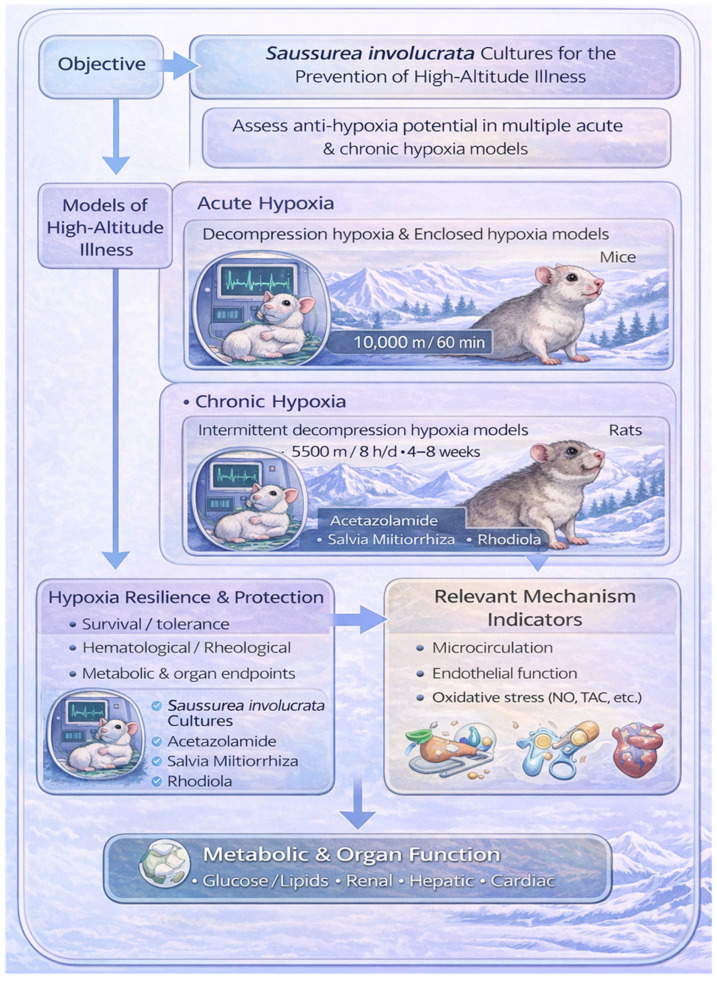
Experimental workflow for investigating the protective effects of SICs against acute and chronic hypoxia-induced damage.

To this end, we designed a stepwise research framework (Figure 1). First, the acute model was employed to verify the fundamental anti-hypoxic tolerance capacity of SICs. Subsequently, the acute injury model was utilized to dissect the regulatory effects of SICs on the hypoxia injury spectrum. Finally, the chronic model was applied to investigate its long-term protective potential. We hypothesize that SICs, produced via a standardized plant cell culture system, can primarily enhance hypoxia tolerance and mitigate hypoxic injury by modulating endothelial function and oxidative stress balance, thereby improving microcirculatory homeostasis. This study focuses on three core pathological axes: endothelial function, oxidative stress, and hemorheology. The findings aim to provide systematic experimental evidence for the scientific characterization of SICs and establish a reference pathway for the standardized translation of natural products in high-altitude medicine.

## 2. Materials and Methods

### 2.1. Materials

Saussurea involucratacultures (20140417) were provided to Shanxi Kangbao Pharmaceutical Co., Ltd. (Changzhi, China). SICs is converted into powder after being harvested and freeze-dried. Before the experiment, the powder is prepared into a suspension of the required concentration with pure water for gavage. Diamox (99.9%) was purchased from Chengdu Youlian Biotech Co., Ltd. (Chengdu, China). Rhodiola (20140417) was obtained from Beijing Tongrentang Lin Pharmaceutical Co., Ltd. (Beijing, China). Compound Danshen Dripping Pills (CDDP, 140416) were sourced from Tianjin Tiantuo Pharmaceutical Group Co., Ltd. (Tianjin, China). Hypoxia reagents kit, Kaomarslan Test Reagents, and Skin Care-1-1 were purchased from Beijing Puer Analytical Co., Ltd. (Beijing, China). Oxygen-activated chemical solutions were purchased from Nanjing Construction Technology Co., Ltd. (Nanjing, China). Formulated Pelleted Feed was obtained from Beijing Huafukang Biotechnology Co., Ltd. (Beijing, China). Laboratory Animal Bedding (Wood Shavings) was obtained from Xinsen Company (Liangfang, China).

Kunming mice (SPF, 50 female/321 male, 18–22 g) and Wistar rats (SPF, 207 male, 180–220 g) were purchased from GemPharmatech Co., Ltd. (Nanjing, China). Environmental conditions were temperature 25 ± 1 °C, humidity 60% ± 5%, a 12 h light–dark cycle with free access to food and water. Mice were housed in groups of 6 to 8 in independent ventilation cages (37 cm  ×  21 cm  ×  14 cm) equipped with environmental enrichment. Wistar rats were housed in groups of 3 to 5 in independent ventilation cages (48 cm  ×  35 cm  ×  20 cm) equipped with environmental enrichment. All animals were individually labeled. Body weight was then used as the main stratification factor. Animals were assigned to each experimental group using a computer-generated random number table. Drug administration, hypoxia exposure, behavioral tests, final sample collection, and index detection were all performed on an individual animal basis. All animal experiments were approved by the Laboratory Animal Ethics Committee in the Institute of Materia Medica and Peking Union Medical College (IMM-S-25-0909).

### 2.2. Evaluation of Acute Anti-Hypoxic Tolerance in Mice

The anti-hypoxic tolerance potential of SICs was preliminarily evaluated using two acute hypoxia models: the acute hypobaric hypoxia model (simulating high-altitude decompression) and the normobaric closed-chamber hypoxia model. Specifically, the protocol for the hypobaric hypoxia model was performed as previously described [14].

#### 2.2.1. Acute Hypobaric Hypoxic Lethality Model (8-Day Administration)

Mice were randomly assigned to five groups (*n* = 20 up, *n* = 10 of each sex). The groups were designated as follows: low-dose SICs (SICs-L, 0.5 g/kg), medium-dose SICs (SICs-M, 1.0 g/kg), high-dose SICs (SICs-H, 1.5 g/kg), CDDP (0.10441 g/kg), and the negative control (Model group). Mice in the Model group received an equivalent volume of sterile water via oral gavage. All groups were administered treatment once daily for 8 consecutive days. One hour after the final administration on day 8, mice were placed in a multi-factor composite environment simulation medical experiment chamber (AVIC Guizhou Fenglei Aviation Ordnance Co., Ltd., Anshun, China). The chamber pressure was reduced to simulate an altitude of 10,000 m (Atmospheric pressure: 26.40 kPa, Oxygen partial pressure: 5.53 kPa) at an equivalent ascent rate of 14–15 m/s. After maintaining this pressure for 60 min, the chamber was repressurized, and the mice were removed. The number of deaths in each group was recorded, and the mortality rate was calculated.

#### 2.2.2. Acute Hypobaric Hypoxic Lethality Model (16-Day Administration)

Preliminary observations indicated significant body weight fluctuations in male mice during the 16-day treatment, a factor known to confound hypoxia tolerance assessment. To ensure a clear interpretation of SICs’ efficacy, the primary analysis was focused on the female cohort, which exhibited stable physiological parameters. Mice were randomly divided into seven groups (*n* = 20 per group). The treatment groups were: SICs-L (0.5 g/kg), SICs-M (1.0 g/kg), SICs-H (1.5 g/kg), Diamox (0.16 g/kg, administered for the final 3 days following 13 days of vehicle treatment), Rhodiola (0.77340 g/kg), CDDP (0.10441 g/kg), and Model group (vehicle control). All groups received daily gavage administration for 16 consecutive days. One hour after the last dose on day 16, mice were exposed to the hypobaric chamber following the identical protocol described in Section 2.2.1 (simulated altitude of 10,000 m, 14–15 m/s ascent rate, maintained for 60 min). Mortality rates were calculated after repressurization.

#### 2.2.3. Closed Hypoxia Endurance Model (3-Day Administration)

Following a 3-day acclimatization period, mice were randomly allocated to five groups (*n* = 10 per group). The groups included: SICs-L (0.5 g/kg), SICs-M (1.0 g/kg), SICs-H (1.5 g/kg), Diamox (0.16 g/kg), and the Model group. Mice were administered treatments once daily for 3 consecutive days. One hour after the final administration on day 3, individual mice were placed into sealed wide-mouth bottles containing soda lime (to absorb CO_2_ and prevent gas accumulation). The time to death (in minutes) was continuously monitored and recorded for each mouse, serving as the index of hypoxic endurance.

### 2.3. Acute Hypoxic Injury Model Establishment and Mechanistic Assays in Rats

As a primary model to dissect early protective mechanisms, male Wistar rats were subjected to acclimatization feeding for 3 days. Then they were randomly divided into 8 groups, with 9 rats per group. The groups included the normoxic control group (Control), hypoxic model group (Model), SICs-L (0.34633 g/kg), SICs-M (0.69266 g/kg), SICs-H (1.03899 g/kg), Diamox group (0.11083 g/kg), Rhodiola group (0.53570 g/kg), and CDDP group (0.07231 g/kg). The Control and Model groups were intragastrically administered with an equal volume of pure water once daily. The remaining groups were given the corresponding doses via intragastric administration once daily for 7 consecutive days. At 1 h after the final administration, the Model group and all drug-treated groups (hypoxic treatment groups) were placed in a hypobaric chamber. The chamber pressure was reduced at an equivalent ascent rate of 10 m/s to simulate an altitude of 5500 m (Atmospheric pressure: 51.86 kPa, Oxygen partial pressure: 10.86 kPa). This condition was maintained for 8 h, followed by a recovery to normal pressure before the rats were taken out. The Control group was not placed in the hypobaric chamber, and all other feeding and treatment conditions were consistent across groups. The body weight of the rats was recorded throughout the experiment, and after hypoxic exposure, the rats were anesthetized. Abdominal aorta blood was collected. The blood samples were centrifuged at 1500× *g* for 15 min using a Heraeus high-speed refrigerated centrifuge (Kendro, Langenselbold, Germany). The serum was separated and stored at −80 °C.

Serum-related indicators were detected using a FlexStation 3 Multi-Mode Microplate Reader (Molecular Devices, Silicon Valley, San Jose, CA, USA). The key mechanistic indicators are endothelial function and oxidative stress. The endothelial function indicators included endothelin-1 (ET-1) and nitric oxide (NO). The oxidative stress indicators included superoxide dismutase (SOD), total antioxidant capacity (TAC), and malondialdehyde (MDA). The glycolipid metabolism indicators included blood glucose, triglycerides, total cholesterol, high-density lipoprotein cholesterol (HDL-C), and low-density lipoprotein cholesterol (LDL-C). The protein metabolism indicators included total protein and albumin. The organ function indicators included alanine aminotransferase (ALT), aspartate aminotransferase (AST), total bilirubin (TBIL), blood urea nitrogen (BUN), creatinine (CR), uric acid (UA), lactate dehydrogenase (LDH), creatine kinase (CK), and α-hydroxybutyrate dehydrogenase (α-HBDH).

### 2.4. Chronic Hypoxic Injury Model in Rats

To evaluate the long-term adaptation and efficacy limitations of SICs’, a chronic intermittent hypoxia model in male Wistar rats was established as the second primary model. Male Wistar rats were acclimatized for 3 days before being randomly assigned to groups, with 9 rats per group (*n* = 9). The basic groups included the normoxic control group (Control), hypoxic model group (Model), SICs-L (0.34633 g/kg), SICs-M (0.69266 g/kg), SICs-H (1.03899 g/kg), and Diamox group (0.11083 g/kg). For the 8-week experiment, additional groups were set up: Rhodiola group (0.53570 g/kg), normoxic diamox group (C-diamox, 0.11083 g/kg), and normoxic SICs medium-dose group (C-SICs-M, 0.69266 g/kg). These groups were used to exclude the baseline effects of the drugs themselves on rats under normoxic conditions.

Drugs were administered daily for 4 or 8 weeks, with daily 8 h exposure to 5500 m hypoxia. The Control group and normoxic control subgroups were not placed in the hypobaric chamber, and all other procedures remained consistent. At the 4-week time point, the right ventricular pressure of the rats was measured using a pressure sensor. Abdominal aortic blood was then collected for routine blood tests, hemorheology tests, and analysis of serum biochemical and mechanistic indicators (the same detection items as in Section 2.3). The 8-week time point was set as the experimental endpoint. Abdominal aortic blood was collected at the endpoint, and on the basis of the indicators tested at 4 weeks, homocysteine (HCY) was added as an additional detection item.

### 2.5. Chronic Hypoxia Model Validation in Mice

To provide cross-species exploratory validation and assess a broader range of functional outcomes, a parallel chronic model was established in male Kunming mice. Male Kunming mice were acclimatized for 3 days. They were then randomly divided into 9 groups, with 9 mice per group (*n* = 9). The groups included the normoxic blank control group (Control), hypoxic model group (Model), SICs-L (0.5 g/kg), SICs-M (1.0 g/kg), SICs-H (1.5 g/kg), Diamox group (0.16 g/kg), Rhodiola group (0.77340 g/kg), normoxic diamox group (C-Diamox, 0.16 g/kg), and normoxic SICs medium-dose group (C-SICs-M, 1.0 g/kg). Mice underwent 8 weeks of daily drug administration and 8 h hypoxia (5500 m).

Given that chronic hypoxia may impair cognitive function, potentially through mechanisms involving microcirculatory disturbance and oxidative stress, we performed the Morris water maze test to exploratively assess whether SICs confers neuroprotective benefits. Training was initiated in the 7th week of hypoxic exposure. The mice were trained twice a day for 3 consecutive days. On the 4th day, the spatial learning and memory abilities were tested, and indicators, including escape latency and total distance traveled, were recorded. At the end of the 8th week, the mice were anesthetized and weighed. Abdominal aortic blood was collected and centrifuged at 3000× *g* for 15 min to separate the serum. Metabolic and organ function analyses were performed.

### 2.6. Research Endpoint

To clarify the research focus, this study defined the main research endpoints based on the core objectives of each model. In the acute mouse hypoxia tolerance model, the primary endpoints were the survival rate after low-pressure hypoxia exposure and the survival time in a confined environment. Acute and chronic hypoxia injury models based on rats were the main models in this study. In the acute hypoxia injury rat model, the primary endpoints focused on the core serum indicators reflecting endothelial function (ET-1, NO) and oxidative stress (MDA, TAC). In the chronic hypoxia injury rat model, the primary endpoints were set as whole blood viscosity, which reflects the microcirculation status, and the levels of serum NO and TAC. In addition, the Morris water maze behavioral indicators in the chronic mouse model were listed as exploratory endpoints. The research conclusions will first be drawn based on the analysis of the above-mentioned primary endpoints, and other detection indicators will serve as important components of the systematic phenotypic description.

### 2.7. Statistical Analysis

All data are presented as mean ± standard deviation (SD). For comparisons among multiple groups, one-way analysis of variance (ANOVA) was first performed. If ANOVA indicated a significant difference (*p* < 0.05), Tukey’s post hoc test was further used for pre-specified pairwise comparisons between groups. All *p*-values for intergroup comparisons reported in this manuscript are derived from this ANOVA + Tukey test procedure. For comparisons involving only two groups, the unpaired *t*-test was used. A *p*-value < 0.05 was considered statistically significant.

## 3. Results

### 3.1. Evaluation of Acute Hypoxia Tolerance in Mice

**Figure 2:**
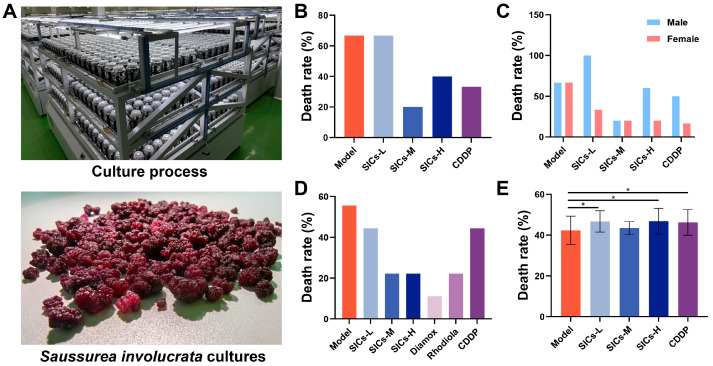
Large-scale culture of SICs and its regulatory effects on acute hypoxia tolerance in mice: (**A**) Large-scale culture process and product morphology of *Saussurea involucrata* cultures. (**B**) Overall mortality of mice in the 8 days after 60 min exposure to a simulated altitude of 10,000 m, *n* = 20. (**C**) Sex-specific mortality of mice in the 8 days after 60 min exposure to a simulated altitude of 10,000 m, *n* = 10. (**D**) Mortality of female mice in the 16-day long-term hypoxia exposure model, *n* = 20. (**E**) Mortality of mice in different groups in the closed-system hypoxia endurance model, *n* = 10, * *p* < 0.05.

The effect of SICs on acute hypoxia tolerance in mice was systematically evaluated using the hypobaric chamber-induced acute hypoxic lethality model (8 and 16 d) and the closed-system hypoxia endurance model. Figure 2A shows the large-scale culture process and final product morphology of SICs. The upper panel displays the large-scale production scenario of cell engineering culture equipment, while the lower panel shows the actual SICs product obtained via culture. This standardized culture system ensures batch-to-batch consistency of the experimental material.

#### 3.1.1. Hypobaric Chamber Lethality Model (8 d)

Under the condition of 60 min exposure to simulated 10,000 m altitude, the mortality rate of mice in the Model group was 66.67% (Figure 2B). Compared with the Model group, the mortality rate of the SICs-M group (1.0 g/kg) significantly decreased to 20.00%, showing the most prominent protective effect. The mortality rates of the SICs-H group (1.5 g/kg) and CDDP group were 40.00% and 33.33%, respectively, both showing a downward trend but not reaching the effect of SICs-M. The mortality rate of the SICs-L group (0.5 g/kg) was close to that of the Model group (66.67%), with no obvious protective effect. Sex-stratified analysis (Figure 2C) showed differences in mortality between male and female mice in the SICs-L group, suggesting that sex-related variability may lead to unstable effects at this dose.

#### 3.1.2. Hypobaric Chamber Lethality Model (16 d)

Since male mice showed significant body weight fluctuations during the 16-day administration period in the preliminary experiment (which might interfere with hypoxia tolerance evaluation), female mice were the main subjects in this section. The results showed that the mortality rate of female mice in the Model group was 55.56% (Figure 2D). Compared with the Model group, the mortality rates of the SICs-M (1.0 g/kg) and SICs-H (1.5 g/kg) groups both decreased to 22.22%, with consistent protective effects. The mortality rate of the SICs-L group (0.5 g/kg) was 44.44%, with a limited protective effect. Among the positive controls, the Diamox group had the lowest mortality rate (11.11%), and the Rhodiola group showed an effect comparable to that of the SICs-M/SICs-H groups (22.22%). The mortality rate of the CDDP group was 44.44%. These results indicate that medium- and high-dose SICs confers stable protection against acute lethal hypoxia in female mice under physiologically stable conditions.

#### 3.1.3. Closed-System Hypoxia Endurance (Survival Time)

This model was used to evaluate the survival tolerance of mice under non-lethal hypoxia conditions. The average survival time of mice in the Model group was 42.34 ± 6.98 min. The survival times of the SICs-L group (0.5 g/kg) and SICs-H group (1.5 g/kg) were significantly prolonged (46.73 ± 5.24 min and 46.86 ± 6.31 min, respectively; *p* < 0.05). The survival time of the SICs-M group (1.0 g/kg) was 43.46 ± 3.13 min, with no statistical difference (*p* > 0.05) (Figure 2E). The survival time of the positive control Diamox group was 46.23 ± 6.29 min (*p* < 0.05), consistent with the effects of the SICs-L/SICs-H groups.

Combining the results of the three acute hypoxia models, SICs significantly enhances acute hypoxic tolerance by both reducing mortality under lethal conditions and prolonging survival under sub-lethal stress. A key observation is that the medium dose (SICs-M) exhibited optimal or consistently stable efficacy in the two lethal hypoxia models. The anti-hypoxic effect of SICs demonstrates clear dose-dependency and notable model-specificity. These findings provide a solid pharmacodynamic basis for subsequent investigations into SICs’ effects on acute hypoxic injury.

### 3.2. Protective Effects and Mechanisms of SICs Against Acute Hypoxic Injury in Rats

This section investigated the acute protective effects of SICs using a rat model exposed to hypobaric hypoxia simulating 5500 m for 8 h. We focused on systemic injury markers and core mechanistic pathways.

#### 3.2.1. General Status of Rats in Acute Hypoxic Injury Model

**Figure 3:**
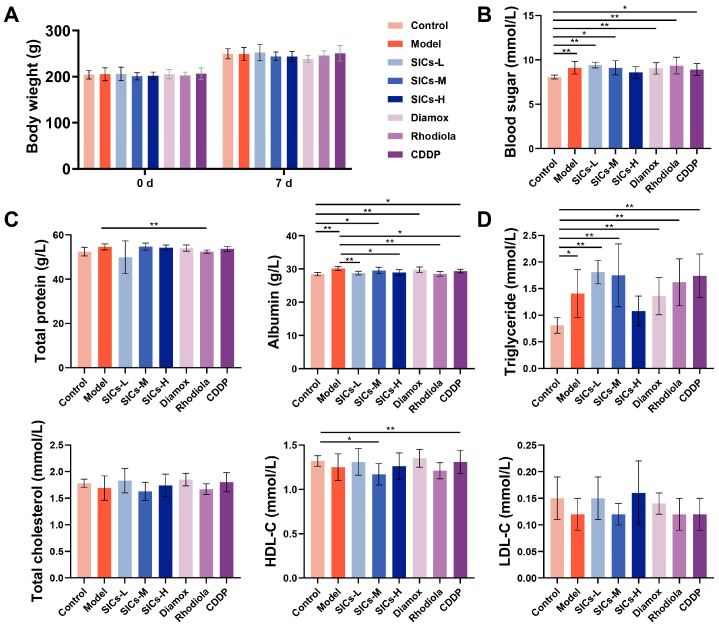
Interventional effects of SICs on body weight and serum metabolic indicators in rats exposed to acute hypoxia (5500 m × 8 h): (**A**) Body weight changes in rats in each group after 7 days of administration, *n* = 9. (**B**) Serum glucose levels, *n* = 9, * *p* < 0.05, ** *p* < 0.01. (**C**) Serum total protein and albumin levels, *n* = 9, * *p* < 0.05, ** *p* < 0.01. (**D**) Serum lipid metabolism-related indicators, including triglycerides, total cholesterol, HDL-C, and LDL-C, *n* = 9, * *p* < 0.05, ** *p* < 0.01.

After 7 consecutive days of administration, the body weight of rats in all groups showed a natural growth trend. No significant difference was observed in the final body weight between the Model group and the Control group (Figure 3A). This indicated that body weight was not a sensitive endpoint for reflecting hypoxic injury under the acute hypoxic exposure window. Notably, the final body weight of the Diamox group was significantly lower than that of other groups, suggesting that Diamox might restrict body weight gain by affecting water–electrolyte metabolism or appetite regulation.

#### 3.2.2. Metabolic Regulation

Metabolic disorders, including abnormalities in blood glucose, protein, and lipid metabolism, are one of the core characteristics of high-altitude hypoxic response. The intervention effect of SICs on the above metabolic indicators under hypoxic conditions showed obvious dose differences. Compared with the Control group, the blood glucose level of the Model group increased significantly (*p* < 0.01) (Figure 3B). After SICs intervention, the blood glucose level of the SICs-H group decreased to a level with no significant difference from the Control group, while the SICs-L and SICs-M groups still maintained hyperglycemia (*p* < 0.01 and *p* < 0.05, respectively). These results suggested that high-dose SICs had a certain tendency to alleviate hypoxia-induced stress hyperglycemia.

In terms of protein metabolism, the total protein level showed small fluctuations without a clear pattern among all groups (Figure 3C). The albumin level of the Model group was significantly higher than that of the Control group (*p* < 0.01). The SICs-L and SICs-H groups could down-regulate the albumin level to be close to that of the Control group, indicating that SICs exerted a selective corrective effect on hypoxia-related protein metabolic disorders.

Among lipid metabolism indicators, the triglyceride level of the Model group was significantly higher than that of the Control group (*p* < 0.05) (Figure 3D). The triglyceride level of the SICs-H group decreased significantly and showed no difference from the Control group (*p* > 0.05). This indicated that the regulation of triglyceride by SICs had a dose-window characteristic, with only the high dose of SICs-H being effective. No significant differences were found in total cholesterol and LDL-C levels among all groups, but the HDL-C levels of the SICs-M group and Rhodiola group were lower than those of the Control group. Acute hypoxia could induce obvious stress hyperglycemia and elevated triglycerides. SICs-H showed a regulatory trend of restoring these two indicators to normoxic levels, but its regulation of lipid metabolism was dependent on the dose window and endpoint selectivity, which required comprehensive judgment based on multiple indicators.

#### 3.2.3. Endothelial Function

**Figure 4:**
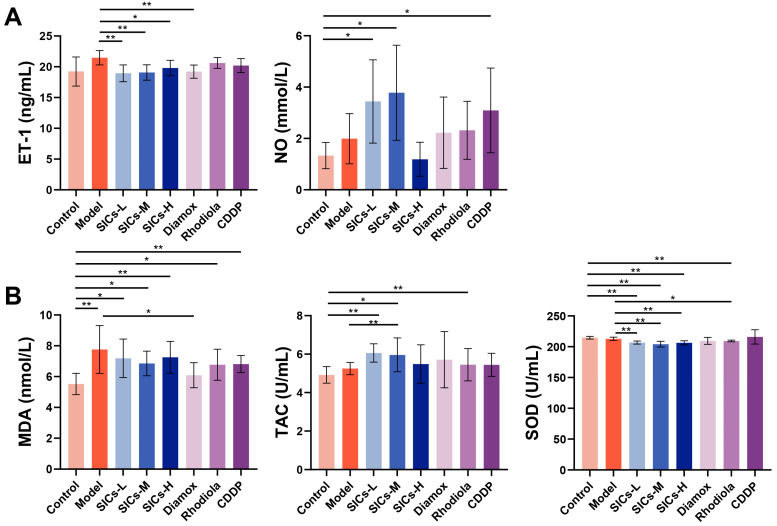
Regulatory effects of SICs on endothelial function and oxidative stress-related indicators in rats exposed to acute hypoxia: (**A**) Core indicators of endothelial function, including ET-1 and NO levels, *n* = 9, * *p* < 0.05, ** *p* < 0.01. (**B**) Oxidative stress-related indicators, including SOD, TAC, and MDA levels, *n* = 9, * *p* < 0.05, ** *p* < 0.01.

Vascular endothelial dysfunction is a key link in high-altitude hypoxic injury. Hypoxia can trigger pathological reactions such as pulmonary vasoconstriction by inducing the imbalance of vasoactive substances and disrupting the balance of vascular contraction and relaxation [15]. As a core endothelium-derived relaxing factor, NO is catalyzed by nitric oxide synthase in endothelial cells, and its level directly reflects the state of endothelial function [16,17]. Although acute hypoxia itself did not significantly alter ET-1 or NO levels versus Control (Figure 4A), SICs treatment markedly modulated this axis. Compared with the Model group, the SICs-L, SICs-M, and SICs-H groups all significantly down-regulated ET-1 (*p* < 0.01, *p* < 0.01, and *p* < 0.05, respectively), and the Diamox group also showed a significant down-regulatory effect (*p* < 0.01). Concurrently, SICs-L and SICs-M significantly increased NO levels above Control, while SICs-H maintained NO at control levels. Although acute hypoxia itself did not cause a significant imbalance of ET-1/NO, SICs could regulate endothelial function through the synergistic effect of down-regulating ET-1 and enhancing NO signaling. This indicated that the regulation of ET-1/NO balance might serve as a core mechanism anchor for endothelial protection by SICs under acute hypoxia.

#### 3.2.4. Oxidative Stress

High-altitude hypoxia can induce oxidative stress injury by causing excessive production of reactive oxygen species, disrupting the redox balance of the body [18,19]. The effect of SICs on oxidative stress indicators in rats is shown in Figure 4B. Compared with the Control group, the MDA levels in the Model group increased significantly (*p* < 0.01), indicating that acute hypoxia could induce enhanced lipid peroxidation. The increase in lipid peroxidation was accompanied by a significant decrease in antioxidant defense [20,21]. The MDA levels in all SICs dose groups remained significantly higher than those in the Control group (*p* < 0.05, *p* < 0.05, and *p* < 0.01, respectively) and did not decrease significantly compared with the Model group (*p* > 0.05). The TAC level in the SICs-L group increased significantly (*p* < 0.01), and the SICs-M group was also higher than the Control group (*p* < 0.05). The SOD activity of all SICs dose groups was significantly lower than that in the Model group (*p* < 0.01). It should be noted that inconsistent changes in different antioxidant endpoints (SOD and TAC) are common under the acute stress window [22]. This might be related to the compensatory regulatory differences between the enzymatic antioxidant system (SOD) and non-enzymatic antioxidant reserves (TAC) during acute hypoxic stress, which is consistent with the characteristics of redox dynamic balance in the early stage of hypoxic stress. Therefore, the evaluation of the antioxidant effect of SICs necessitates a comprehensive interpretation based on multiple indicators such as MDA and TAC, rather than solely relying on enzymatic indicators. The finding that SICs could increase TAC suggested that it tended to enhance the antioxidant reserve/capacity of the body, rather than directly scavenging the already–produced peroxidation products.

#### 3.2.5. Organ Function

High-altitude hypoxia can interfere with liver metabolic homeostasis, leading to abnormal liver enzyme activity and bilirubin metabolism disorders [23,24]. The results showed (Figure S1A) that the AST and TBIL levels of the Model group increased significantly (*p* < 0.05). The AST levels of all SICs dose groups were still significantly higher than those of the Control group (*p* < 0.01), and the TBIL levels did not show consistent improvement, while the Diamox group could significantly reduce AST (*p* < 0.05). No significant difference was found in ALT among all groups, suggesting that the liver injury caused by acute hypoxia was more concentrated on AST-related metabolic stress, and the reversal effect of SICs on this injury was limited.

High-altitude hypoxia can reduce renal blood oxygen perfusion, which may induce compensatory or injurious changes in renal function. The results of renal function detection showed (Figure S1B) that there were no significant differences in BUN, CR, and UA levels between the Model group and the Control group. However, the BUN levels of all SICs dose groups increased significantly (*p* < 0.05, *p* < 0.01, and *p* < 0.01, respectively), and only the UA level of the SICs-M group showed no difference from both the Control group and the Model group. As the final product of purine metabolism, UA has both oxidizing and antioxidant properties [25]. The above results indicated that the fluctuations of renal function-related indicators had no clear pattern within the acute hypoxia window, and the effect of SICs on renal function needed further verification of direction and persistence in chronic models.

High-altitude reaction can induce myocardial injury by increasing cardiac load [26]. The results of myocardial enzyme spectrum detection showed (Figure S1C) that the α-HBDH level of the Model group increased significantly (*p* < 0.05), while LDH and CK showed no significant differences. The Diamox group and Rhodiola group could significantly reduce LDH (*p* < 0.05), while SICs had no clear regulatory pattern on the myocardial enzyme spectrum. In summary, the organ-level injury caused by acute hypoxia was more concentrated in the elevation of AST, TBIL, and the abnormality of α-HBDH, while the corrective effect of SICs on the above-mentioned liver bilirubin metabolism disorders and myocardial enzyme spectrum disorders was unstable.

Acute hypobaric hypoxia could induce a systemic injury profile in rats, characterized by stress hyperglycemia, elevated triglycerides, enhanced lipid peroxidation, and abnormal indicators related to liver, heart, and kidney injuries. Within this window, SICs exhibited a selective mechanism-regulating effect. SICs could regulate endothelial function by downregulating ET-1 and enhancing NO signaling, and enhance antioxidant reserves by increasing TAC, but its reversal effect on MDA and organ function abnormalities (liver, heart, and kidney) was limited. The subsequent research will focus on the long-term regulatory effect of SICs on the pathological chain of hyperviscosity–microcirculation disorder/endothelial function–oxidative stress–organ load and the optimal dose window.

### 3.3. Protection Against Chronic High-Altitude Hypoxic Injury in Rats

This section systematically explored the long-term interventional effects of SICs on chronic high-altitude hypoxic injury in rats using hypoxic models (simulated altitude of 5500 m, 8 h daily exposure, lasting 4 weeks [subchronic] and 8 weeks [chronic]).

#### 3.3.1. General Status and Body Weight

The macroscopic phenotypic changes induced by chronic hypoxic exposure were mainly reflected in body weight gain and organ load remodeling. The body weight gain rate of Model rats slowed significantly from the 2nd week onward, and this trend persisted until the 8-week experimental endpoint (Figure S2), indicating that long-term hypoxia inhibits body growth and development. Organ index analysis showed that the heart and spleen indices of the Model group were significantly increased (*p* < 0.01) (Figure S3). This is speculated to be due to compensatory hyperplasia in response to hypoxia, such as the compensation of cardiac pumping function and the activation of splenic hematopoietic function. In contrast, the kidney index significantly decreased, which may be related to hypoxic renal hypoperfusion. None of the SICs doses significantly reversed the suppressed weight gain or markedly normalized these organ indices, indicating limited effects on macroscopic hypoxia-induced remodeling.

#### 3.3.2. Hemodynamics and Hemorheology

**Figure 5:**
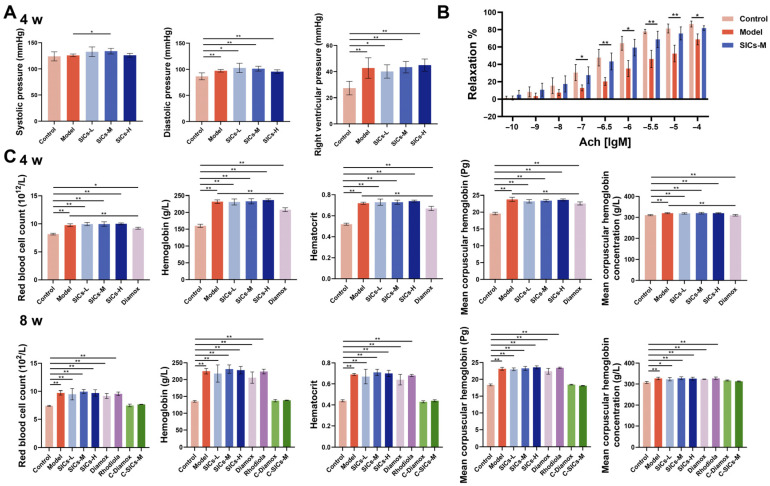
Interventional effects of SICs on blood pressure and hematological indicators in rats under chronic hypoxic exposure (4 and 8 weeks): (**A**,**B**) Right ventricular pulmonary pressure and diastolic blood pressure levels, *n* = 9, * *p* < 0.05, ** *p* < 0.01. (**C**) Core hematological indicators, including red blood cell count, hemoglobin, and hematocrit, *n* = 9, * *p* < 0.05, ** *p* < 0.01.

High-altitude hypoxia can induce pulmonary vasoconstriction, leading to pulmonary hypertension and further increasing the pulmonary circulatory load [27,28]. Chronic hypoxic exposure (4 and 8 weeks) significantly disrupted rat circulatory function, and was specifically manifested by the significant increases in right ventricular systolic pressure (a core indicator of pulmonary circulatory pressure) and diastolic pressure in the Model group (Figure 5A,B), indicating a continuous increase in pulmonary circulatory load. None of the SICs dose groups significantly reduced right ventricular systolic pressure, suggesting that SICs had no significant reversal effect on chronic hypoxia-induced pulmonary hypertension.

Chronic hypoxic exposure can induce hematological changes similar to high-altitude polycythemia in rats [29]. Specifically, the red blood cell count, hemoglobin content, and hematocrit in the Model group significantly increased (Figure 5C), and blood viscosity was significantly elevated at multiple shear rates (Figure S4). This indicates that the body had developed a hyperviscous state, which may exacerbate microcirculatory disorders. The regulation of hemorheology by SICs showed a clear dose dependence. The SICs-L and SICs-M groups had no significant effect on blood viscosity, while the SICs-H group significantly reduced blood viscosity, with a more pronounced improvement effect at low shear rates. These results suggest that SICs-H has a targeted improvement effect on hypoxia-induced microcirculatory rheological abnormalities. Overall, SICs mainly regulates hemorheology at the microcirculatory level to improve vascular function injury caused by hypoxia. However, their inhibitory effect on polycythemia is limited, indicating that their protective effect is more focused on maintaining microcirculatory homeostasis rather than directly regulating hematopoietic function.

#### 3.3.3. Endothelial Function and Oxidative Stress

Endothelial dysfunction and oxidative stress imbalance are core mechanisms of chronic hypoxic injury. The interventional effect of SICs on the above indicators is significantly time-window dependent and dose-selective.

**Figure 6:**
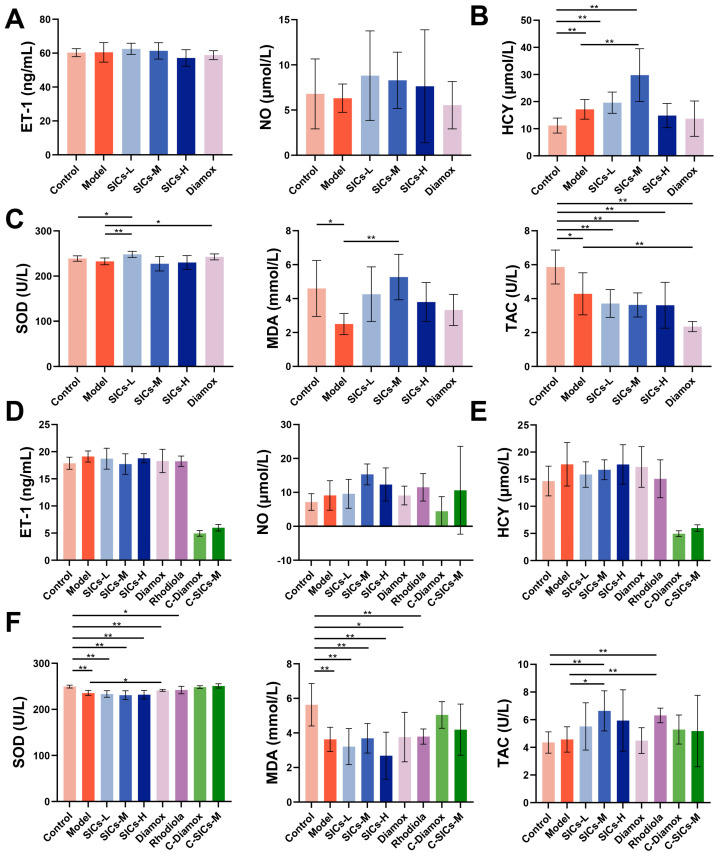
Time-dependent regulatory effects of SICs on endothelial function, homocysteine (HCY), and oxidative stress indicators in rats under chronic hypoxic exposure (4 and 8 weeks): (**A**) ET-1 and NO levels after 4 weeks of chronic hypoxic exposure, *n* = 9. (**B**) HCY levels after 4 weeks of chronic hypoxic exposure, *n* = 9, ** *p* < 0.01. (**C**) Oxidative stress indicators (SOD, MDA, and TAC) after 4 weeks of chronic hypoxic exposure, *n* = 9, * *p* < 0.05, ** *p* < 0.01. (**D**) ET-1 and NO levels after 8 weeks of chronic hypoxic exposure, *n* = 9. (**E**) HCY levels after 8 weeks of chronic hypoxic exposure, *n* = 9. (**F**) Oxidative stress indicators (SOD, MDA, and TAC) after 8 weeks of chronic hypoxic exposure, *n* = 9, * *p* < 0.05, ** *p* < 0.01.

At the 4-week feeding stage, there were no significant differences in the core endothelial function indicators (ET-1/NO) among all groups (Figure 6A). HCY, a marker of vascular endothelial injury, was significantly higher in the Model, SICs-L, and SICs-M groups than in the Control group, while the HCY levels in the SICs-H and Diamox groups decreased to a level not significantly different from the Control group (Figure 6B). This suggests that SICs-H can alleviate the risk of endothelial injury induced by subchronic hypoxia. Oxidative stress indicators showed inconsistent trends: SOD activity was upregulated in the SICs-L group, MDA content increased in the SICs-H group, and TAC significantly decreased in all groups (Figure 6C). These changes reflect compensatory dynamic fluctuations in the antioxidant system during the subchronic hypoxia stage, which is consistent with the characteristics of redox balance during the transition from acute to chronic hypoxia.

At the 8-week feeding stage, there were no significant differences in ET-1 and HCY among all groups (Figure 6D,E), suggesting that long-term hypoxia may bring endothelial-related injuries into a plateau phase or reduce the sensitivity of these indicators to interventions. In contrast, the NO levels in the SICs-M, SICs-H, and Rhodiola groups significantly increased, and only the SICs-M group showed a further increase in NO level compared with the Model group (Figure 6D). This indicates that SICs-M and SICs-H can improve chronic hypoxic vasospasm by enhancing NO-mediated vasodilation function. Regarding oxidative stress, the SOD activity of the Model group significantly decreased, and significantly increased SOD activity was only observed in the Diamox group. TAC significantly increased in the SICs-M and Rhodiola groups (Figure 6F). The stable protective mechanism of SICs during chronic hypoxia concentrated on NO upregulation and TAC enhancement, while their regulatory effects on ET-1, HCY, and SOD were not significant. The dynamic fluctuations and directional heterogeneity of antioxidant indicators suggest that the evaluation of the antioxidant effects of SICs requires comprehensive interpretation based on multiple indicators rather than relying solely on enzymatic indicators.

#### 3.3.4. Metabolism and Organ Function in Rats

Chronic high-altitude hypoxia can have persistent effects on body metabolism and multi-organ function, with significant individual heterogeneity in injuries [30]. The SICs intervention showed clear selective regulatory characteristics.

**Figure 7:**
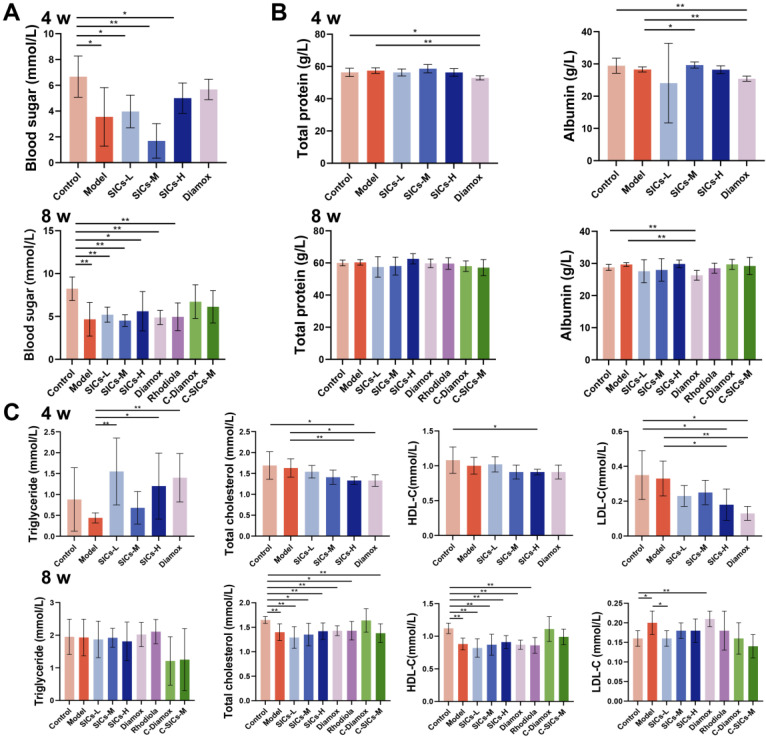
Selective regulatory effects of SICs on metabolism- and organ function-related indicators in rats under chronic hypoxic exposure: (**A**) Blood glucose levels in rats after 4 and 8 weeks of chronic hypoxic exposure, *n* = 9, * *p* < 0.05, ** *p* < 0.01. (**B**) Total protein and albumin levels after 4 and 8 weeks of chronic hypoxic exposure, *n* = 9, * *p* < 0.05, ** *p* < 0.01. (**C**) Lipid metabolism-related indicators, including total cholesterol, HDL-C, and LDL-C, after 4 and 8 weeks of chronic hypoxic exposure, *n* = 9, * *p* < 0.05, ** *p* < 0.01.

At the metabolic level, chronic hypoxia mainly induced glycolipid metabolism disorders and protein metabolism abnormalities. The Model group showed a significant decrease in blood glucose at 8 weeks (Figure 7A), suggesting that long-term hypoxia may inhibit glucose metabolism efficiency. SICs had a certain improvement effect on total protein and albumin levels (Figure 7B), indicating that they can partially alleviate hypoxia-related protein synthesis/metabolism disorders. Lipid metabolism disorders were manifested by a significant decrease in total cholesterol and HDL-C, and a significant increase in LDL-C in the Model group (Figure 7C). A decrease in HDL-C impairs lipid clearance capacity and increases cholesterol accumulation [31], while an increase in LDL-C increases the risk of vascular lipid deposition. At certain doses, SICs can restore the above lipid metabolism indicators to normal levels, showing potential for selective regulation.

At the organ function level, SICs did not show a consistent recovery effect on liver and kidney function. Within the 4- and 8-week time windows, none of the SICs dose groups comprehensively improved liver function indicators (Figure S5A) and renal function indicators (Figure S5B), with only a weak regulatory trend for individual doses on single indicators. Regarding myocardial function protection, SICs showed certain targeting. In the 8-week model, the hypoxia-induced increases in LDH and CK levels could be significantly improved by different SICs doses (Figure S5C), but there was no clear regulatory effect on α-HBDH. This suggests that its repair effect on chronic hypoxic myocardial injury is limited.

Chronic high-altitude hypoxia induces multi-system injuries in rats, such as macroscopic growth inhibition, organ load remodeling, pulmonary hypertension, hyperviscous blood, endothelial dysfunction, oxidative stress imbalance, glycolipid metabolic disorders, and multi-organ dysfunction. The protective effect of SICs against chronic high-altitude hypoxic injury focuses on the core pathological chain of microcirculatory homeostasis–endothelial function–oxidative stress. Its selective regulatory characteristics suggest that it is more suitable for the prevention and treatment of injuries related to microcirculatory disorders, endothelial dysfunction, and oxidative stress imbalance during the long-term high-altitude acclimatization period. This provides a novel potential strategy and experimental basis for the targeted intervention of high-altitude reactions.

### 3.4. Validation of Chronic Hypoxia Model in Mice

This section validated the interventional effect of SICs on chronic hypoxic injury using an 8-week chronic hypobaric hypoxia model in mice.

#### 3.4.1. General Status of Mice in Chronic Hypoxia Model

**Figure 8:**
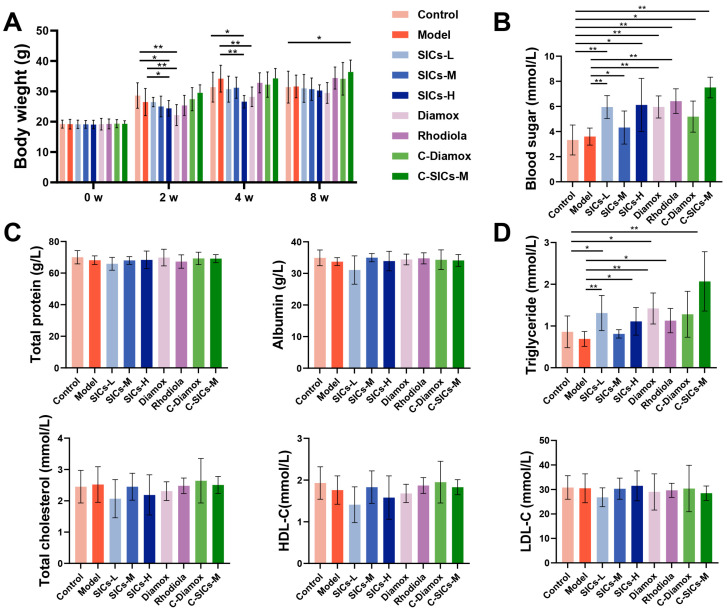
Effects of SICs on body weight and metabolism-related indicators in mice after 8 weeks of chronic hypoxic exposure: (**A**) Body weight growth curves of mice in each group after 8 weeks of chronic hypoxic exposure, *n* = 9, * *p* < 0.05, ** *p* < 0.01. (**B**) Blood glucose levels after 8 weeks of chronic hypoxic exposure, *n* = 9, * *p* < 0.05, ** *p* < 0.01. (**C**) Protein metabolism indicators (total protein, albumin) after 8 weeks of chronic hypoxic exposure, *n* = 9. (**D**) Lipid metabolism indicators (triglycerides, total cholesterol, HDL-C, LDL-C) after 8 weeks of chronic hypoxic exposure, *n* = 9, * *p* < 0.05, ** *p* < 0.01.

During the 8-week experimental period, the body weight of mice in all groups showed a natural growth trend with age (Figure 8A). No significant difference was observed in the final body weight between the Model and the Control group. However, the Model group exhibited phasic fluctuations in body weight gain from Week 2 to Week 4. This indicates that chronic hypoxia affects mice’s body weight more by interfering with the short-term growth rhythm rather than inhibiting long-term growth. Compared with the Model group, the SICs-H and Diamox groups showed a phasic lower body weight at Week 2 and Week 4. The body weight of other groups (SICs-L, SICs-M, and Rhodiola) had limited overall differences from the Model group. These results suggest that some interventions may have a slight impact on the short-term growth of mice, but did not alter the long-term body weight gain trend.

#### 3.4.2. Metabolism and Organ Function in Mice

The effects of chronic hypoxia on mice’s metabolism and organ function were characterized by scattered indicators and fluctuations without a unified pattern. The SICs interventional effect also showed strong endpoint selectivity.

In terms of glucose metabolism, there was no significant difference in blood glucose between the Model and the Control group. However, under hypoxic conditions, the blood glucose levels in the SICs-L, SICs-H, Diamox, and Rhodiola groups were significantly increased (Figure 8B). This suggests that the interventions themselves may regulate glucose metabolism under hypoxia, rather than simply reversing hypoxic injury. Regarding protein metabolism, there were no clear differences in total protein and albumin among all groups (Figure 8C), indicating that chronic hypoxia had a weak impact on mice’s protein metabolism. For lipid metabolism, no significant intergroup differences were found in total cholesterol and HDL-C, and triglyceride levels were significantly increased in the SICs-L and Diamox groups, also showing an increasing trend in the C-SICs-M group. LDL-C only exhibited mild fluctuation in the SICs-M group (Figure 8D), with no unified pattern in lipid metabolism changes.

In terms of organ function, there were no intergroup differences in liver function indicators (ALT and AST). However, the TBIL level of the Model group was increased, and this increase was further enhanced in the SICs-M group. The TBIL levels of the SICs-H, Diamox, and Rhodiola groups also showed an increasing trend (Figure S6A). Fluctuations in renal function indicators (BUN and CR) were limited; only the UA level of the SICs-M group was decreased, and the reduction was more significant in the Diamox group (Figure S6B). Among myocardial enzyme spectrum indicators, the LDH and CK levels of the Model group showed an increasing trend, while the SICs-L, SICs-H, and Diamox groups could reduce CK (Figure S6C). In addition, no intergroup difference was observed in serum HCY.

In the mouse chronic hypoxia model, the signs of organ injury and metabolic disorders were more scattered. Although SICs improved individual indicators such as UA and CK, they were accompanied by increases in blood glucose, triglycerides, and TBIL. These results suggest that the systemic interventional effect of SICs in the mouse chronic hypoxia model is relatively complex, lacking a consistent protective trend.

#### 3.4.3. Behavior (Morris Water Maze)

**Figure 9:**
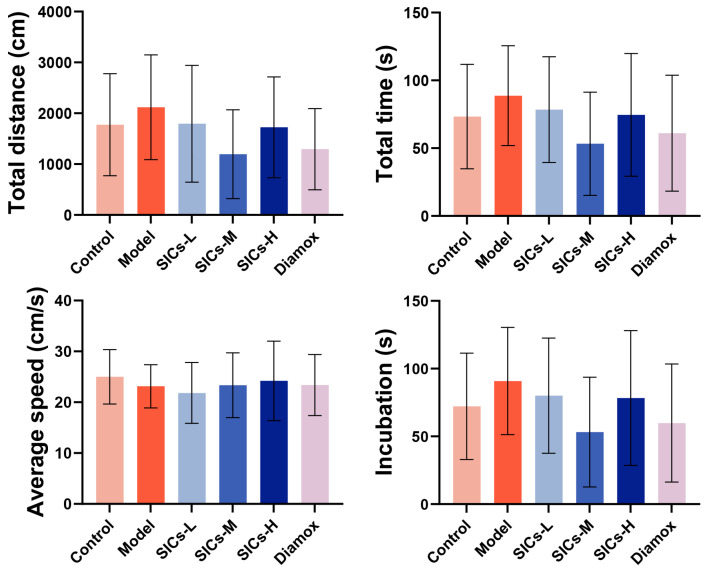
Effects of SICs on Morris water maze behavioral indicators in mice under chronic hypoxic exposure, including total distance, total time, average speed, and escape latency, *n* = 9.

Chronic hypoxia can significantly impair cognitive function, especially spatial learning and memory abilities [32,33]. This study evaluated the potential of SICs to improve such injuries using the Morris water maze. The results showed that the total distance traveled, total time, and escape latency of the Model group tended to be higher than those of the Control group, while the average speed was decreased (Figure 9). This indicates that chronic hypoxia may reduce the spatial learning efficiency and memory retention ability of mice.

The total distance traveled, total time, and escape latency of the SICs-M and Diamox groups showed a decreasing trend, indicating a tendency of cognitive improvement compared with the Model group. However, these differences did not reach statistical significance, which suggests that the improvement effect of SICs-M on chronic hypoxia-induced cognitive impairment is weak, due to a lack of strong evidence.

In the 8-week chronic hypobaric hypoxia mouse model, hypoxia had mild effects on body weight and most protein metabolism indicators. However, it also induced fluctuations in some metabolic and organ function indicators, as well as exerting an adverse trend on behavioral performance related to spatial learning and memory. The interventional effect of SICs showed strong endpoint selectivity, and although they exhibited improvements in individual indicators (CK and UA) and cognitive behavior, these were accompanied by metabolic fluctuations, including increased blood glucose and triglycerides. The overall systemic effect was complex and lacked consistency.

## 4. Discussion

Acute and chronic injuries induced by high-altitude hypoxia constitute a complex systemic pathological process. They involve adaptive and decompensatory responses at multiple levels, including oxygen sensing, metabolic reprogramming, vascular regulation and redox homeostasis imbalance. At present, except for phased acclimatization and a few chemical drugs, safe, convenient and stable preventive intervention strategies are still lacking [9]. In this study, a substance with highly uniform chemical composition (SICs) was obtained via plant cell engineering technology. For the first time, we systematically evaluated its protective potential against high-altitude hypoxic injury in multi-level animal models. The results showed that SICs significantly enhanced the body’s acute tolerance to extreme hypoxia. In both acute and chronic hypoxia models, it exerted a protective effect by selectively regulating the core pathological axis of endothelial function, oxidative stress and hemorheology. Moreover, when used to treat rats/mice, it exhibited a distinct dose-dependent window effect.

The protective effect of the SICs did not exert extensive reversal effects on all hypoxic phenotypes, but precisely targeted the key processes maintaining microcirculation and redox homeostasis. Under acute hypoxia exposure, the SICs synergistically regulated the balance of vasoactive substances, as evidenced by the significant downregulation of ET-1 and upregulation of NO levels. This may alleviate abnormal vasoconstriction in the early stage of hypoxia. Meanwhile, the SICs markedly increased serum TAC, indicating that they enhanced the body’s endogenous antioxidant reserves, rather than merely neutralizing already formed oxidative products. This effect was particularly important during the chronic acclimatization phase. Eight weeks of chronic hypoxia induced a typical state of high blood viscosity, whereas the SICs effectively reduced whole blood viscosity, particularly in improving blood flow characteristics at low shear rates. Combined with the sustained elevation in NO levels in the SICs-M and SICs-H groups, these effects jointly targeted microcirculatory perfusion disorders, a critical pathological feature of chronic mountain sickness. Notably, the above-mentioned core effects of SICs exhibited a distinct dose-dependent manner. The medium dose showed optimal or stable efficacy for most key indicators. This dose–response relationship may stem from the complexity of its multi-component composition: at specific doses, various active ingredients (e.g., phenylpropanoids and flavonoids) may reach an optimal ratio to synergistically activate protective pathways (e.g., the eNOS-NO pathway). Deviation from this window may lead to effect saturation or trigger different regulatory responses; this phenomenon was also observed in another study [34]. The clarification of this quantitative relationship represents a key advantage of standardized production over traditional herbal raw materials, providing precise pharmacodynamic evidence for subsequent applications.

In the study design, we integrated multi-time-scale models ranging from acute tolerance to chronic acclimatization. The aim was to comprehensively delineate the action spectrum of SICs. We clarified that the acute and chronic injury models established for rats served as the primary system for dissecting the core mechanisms. As an exploratory extension, we observed the effect of SICs on hypoxia-related cognitive function in a chronic mouse model. Preliminary data indicated a tendency toward improvement in the Morris water maze test for the SICs-treated group. This finding provides preliminary, yet cautiously interpretable. evidence for the hypothesis that hypoxia may impair cognitive function by affecting the intracerebral microenvironment, and that the SICs may confer neural benefits by systematically improving vascular and redox homeostasis. Further studies are required to directly detect the relevant molecular changes in brain tissues, thereby establishing a definitive correlation.

This study has several limitations. First, although the sample size was designed with reference to conventional pharmacodynamic studies in the field and was sufficient to support trend analysis for the primary endpoints, its scale remains within the scope of preclinical screening studies. Future confirmatory studies need to perform more rigorous a priori sample size calculations based on the effect sizes revealed in this study. Second, to reduce bias, we implemented blinding during sample detection and data analysis. However, complete blinding could not be achieved in essential procedures such as animal drug administration, which is an inherent challenge in experimental pharmacology research. Third, significant gender differences were observed in the long-term model. Sex hormones (e.g., estrogen) can affect hypoxia acclimatization by regulating vascular endothelial function, antioxidant enzyme activity, and energy metabolism pathways [35,36]. We mainly analyzed female data for the sake of data stability. Although this facilitated clear interpretation of drug efficacy, it limited the generalizability of the conclusions to the male population. It also suggests that sex hormone background may be a key variable affecting the efficacy of the SICs, which needs to be specifically designed for in future studies. Finally, although the multi-level animal models provided systematic evidence, they still differ from the complex environment of actual high-altitude exposure in humans. The clinical translation effect of the SICs should be verified in prospective population-based studies.

## 5. Conclusions

This study systematically demonstrates the potential of a standardized SICs as a candidate intervention for high-altitude illness. Using a multi-tiered animal model approach, we show that the SICs provides a dual benefit: it enhances tolerance to acute, lethal hypoxia and selectively regulates the core pathological axis of endothelial function, oxidative stress, and hemorheology during sustained hypoxic exposure. The protective effects are mediated through key mechanisms, including the downregulation of ET-1, enhancement in NO-mediated endothelial function, improvement in microcirculatory blood flow, and an increase in TAC. The SICs exhibits a clear dose–response relationship, with medium doses often showing optimal efficacy. Notably, its effects are more pronounced in these specific mechanistic pathways than in reversing macroscopic organ remodeling. This pharmacological profile suggests that the SICs is better suited for preventive protection prior to high-altitude exposure or for early-stage intervention, rather than as a treatment for severe altitude sickness. We acknowledge that improvements in some organ function indicators were inconsistent, and inherent differences exist between animal models and human high-altitude exposure. Future studies should focus on validating these findings in human cohort studies and refining the dose-altitude-exposure adaptation strategy. In summary, this research provides a systematic preclinical foundation for the development of SICs and offers a translational framework for the standardized evaluation of natural products.

## Data Availability

The data that support the findings of this study are available in the manuscript. Extra data used to support this study are available from the corresponding author upon request.

## Supplementary Materials

The following supporting information can be downloaded at https://www.mdpi.com/article/10.3390/nu18040556/s1, Figure S1. Effects of SICs on serum indicators related to liver, kidney, and cardiac functions in rats exposed to acute hypoxia: (A) Liver function indicators, including AST, ALT, and TBIL, *n* = 9, * *p* < 0.05, ** *p* < 0.01. (B) Kidney function indicators, including SUN, CR, and UA, *n* = 9, * *p* < 0.05, ** *p* < 0.01. (C) Myocardial injury-related indicators, including α-HBDH, LDH, and CK, *n* = 9, * *p* < 0.05, ** *p* < 0.01. Figure S2. Body weight growth curves of rats under chronic hypoxic exposure (4 and 8 Weeks), *n* = 9, * *p* < 0.05, ** *p* < 0.01. Figure S3. Spleen index, Heart index, and Kidney index in rats under chronic hypoxic exposure (4 and 8 weeks), *n* = 9, * *p* < 0.05, ** *p* < 0.01. Figure S4. Changes in Blood Viscosity at Different Shear Rates in Rats Under Chronic Hypoxic Exposure (4/8 weeks): (A) Blood viscosity at different shear rates (1, 3, 30, 100, 180, and 200/s) after 4 weeks of chronic hypoxic exposure, *n* = 9, * *p* < 0.05, ** *p* < 0.01. (B) Blood viscosity at different shear rates (5, 10, 30, 50, 100, 150, and 200/s) after 8 weeks of chronic hypoxic exposure, *n* = 9, * *p* < 0.05, ** *p* < 0.01. Figure S5. Selective regulatory effects of SICs on liver, kidney, and myocardial function-related indicators in rats under chronic hypoxic exposure (4 and 8 weeks): (A) Liver function indicators (ALT, AST, and TBIL) after 4/8 weeks of chronic hypoxic exposure, *n* = 9, * *p* < 0.05, ** *p* < 0.01. (B) Kidney function indicators (BUN, CR, and UA) after 4/8 weeks of chronic hypoxic exposure, *n* = 9, * *p* < 0.05, ** *p* < 0.01. (C) Myocardial enzyme spectrum indicators (LDH, CK, and α-HBDH) after 4 and 8 weeks of chronic hypoxic exposure, *n* = 9, * *p* < 0.05, ** *p* < 0.01. Figure S6. Effects of SICs on organ function-related indicators in mice after 8 weeks of chronic hypoxic exposure: (A) Liver function indicators (ALT, AST, and TBIL) after 8 weeks of chronic hypoxic exposure, *n* = 9, * *p* < 0.05, ** *p* < 0.01. (B) Kidney function indicators (BUN, CR, and UA) after 8 weeks of chronic hypoxic exposure, *n* = 9, * *p* < 0.05, ** *p* < 0.01. (C) Myocardial enzyme spectrum indicators (LDH, CK, and α-HBDH) after 8 weeks of chronic hypoxic exposure, *n* = 9, * *p* < 0.05, ** *p* < 0.01.

## Abbreviations

The following abbreviations are used in this manuscript:

α-HBDH	α-hydroxybutyrate dehydrogenase
ALT	Alanine aminotransferase
ANOVA	Analysis of variance
AST	Aspartate aminotransferase
BUN	Blood urea nitrogen
CDDP	Compound Danshen Dripping Pills
C-Diamox	Normoxic diamox group
C-SICs-M	Normoxic SICs medium-dose group
CK	Creatine kinase
CR	Creatinine
ET-1	Endothelin-1
HDL-C	High-density lipoprotein cholesterol
HCY	Homocysteine
LDL-C	Low-density lipoprotein cholesterol
LDH	Lactate dehydrogenase
MDA	Malondialdehyde
NO	Nitric oxide
SICs	*Saussurea involucrate* cultures
SICs-H	High-dose SICs
SICs-L	Low-dose SICs
SICs-M	Medium-dose SICs
SOD	Superoxide dismutase
TAC	Total antioxidant capacity
TBIL	Total bilirubin
UA	Uric acid
