# Supplementary material for: Saussurea involucrata Cultures for High-Altitude Illness: Enhancing Hypoxia Tolerance and Protecting Against Acute/Chronic Hypoxic Injury
Source: Nutrients. 2026 Feb 7;18(4):556. doi: 10.3390/nu18040556 (PMC12942915; doi:10.3390/nu18040556)
Supplement: Supplementary file 1 [file nutrients-18-00556-s001.zip › nutrients-4110977-supplementary.pdf]

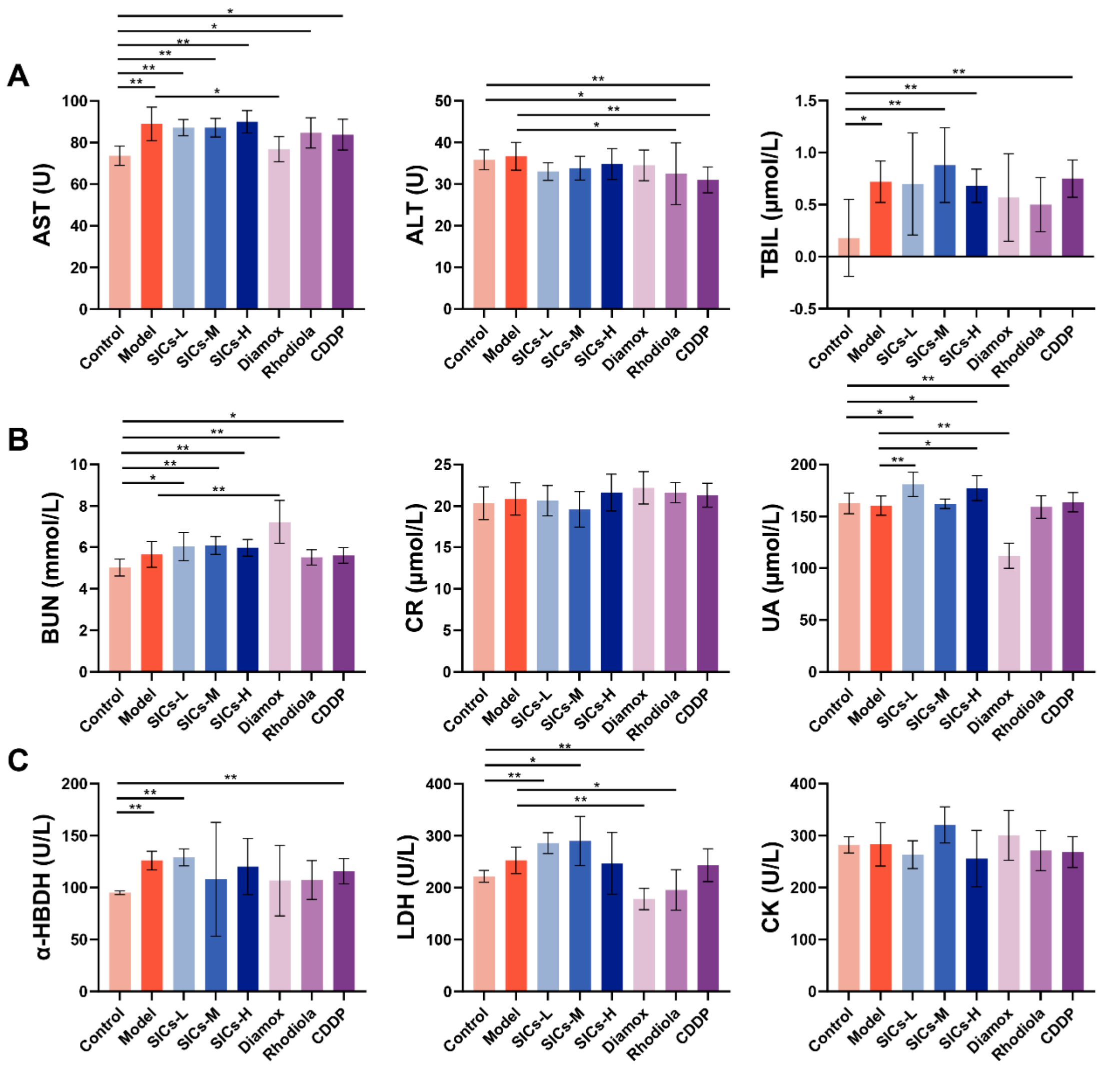

Figure S1. Effects of SIC on serum indicators related to liver, kidney, and cardiac functions in rats exposed to acute hypoxia. (A) Liver function indicators, including AST, ALT, and TBIL, n = 9, * P < 0.05, ** P < 0.01. (B) Kidney function indicators, including SUN, CR, and UA, n = 9, * P < 0.05, ** P < 0.01. (C) Myocardial injury-related indicators, including α-HBDH, LDH, and CK, n = 9, * P < 0.05, ** P < 0.01.

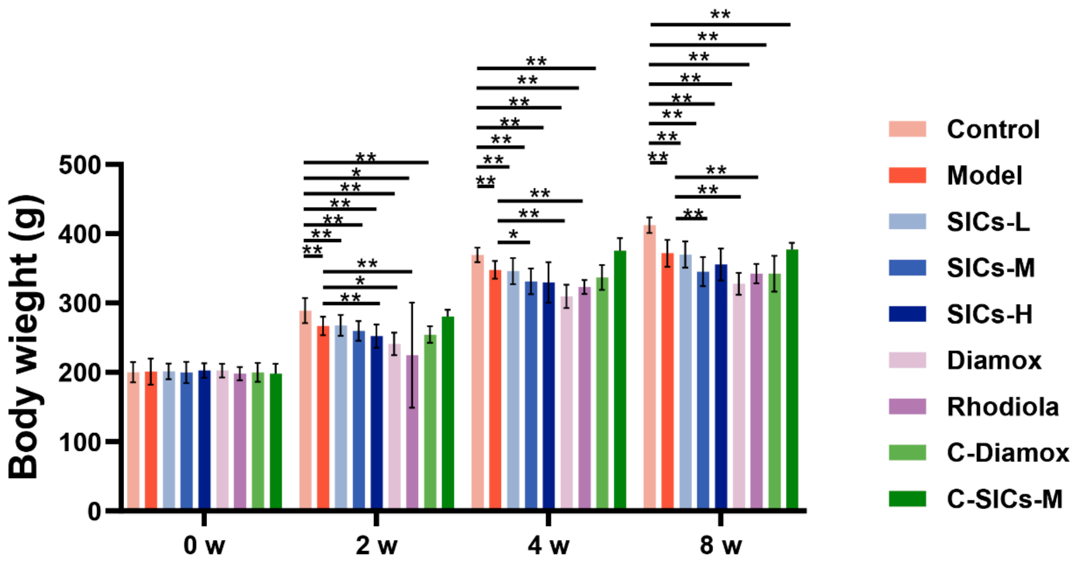

Figure S2. Body weight growth curves of rats under chronic hypoxic exposure (4 and 8 Weeks), n = 9, * P < 0.05, ** P < 0.01.

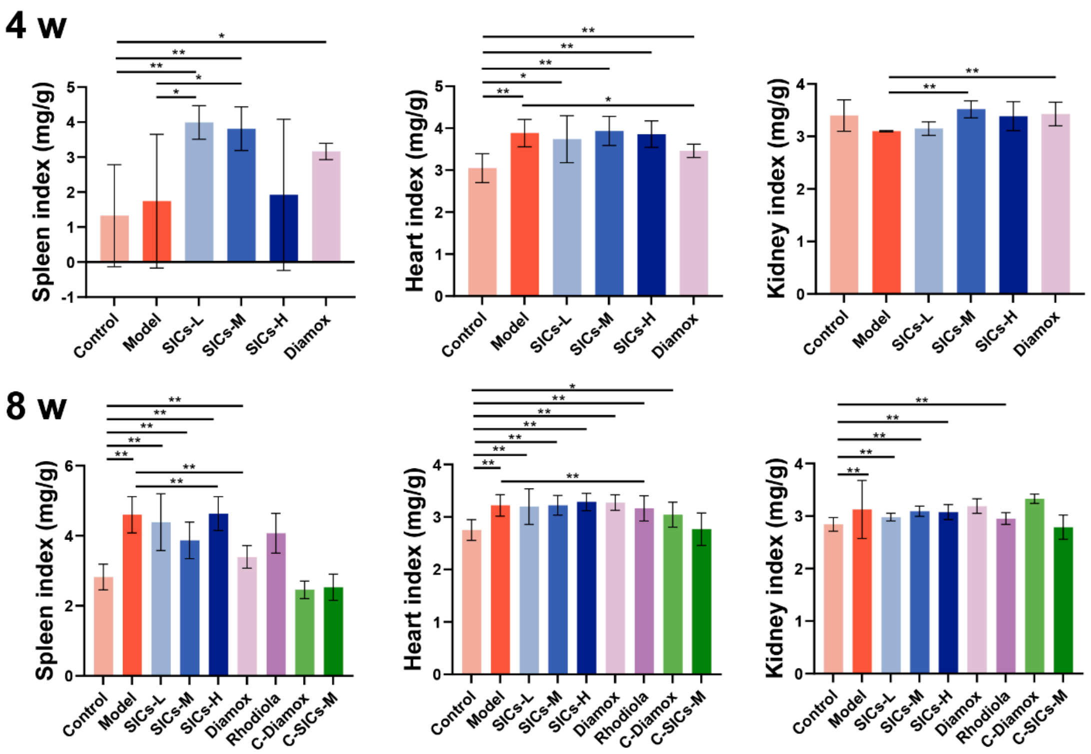

Figure S3. Spleen index, Heart index, and Kidney index in rats under chronic hypoxic exposure (4 and 8 Weeks)., n = 9, * $P < 0.05$, ** $P < 0.01$.

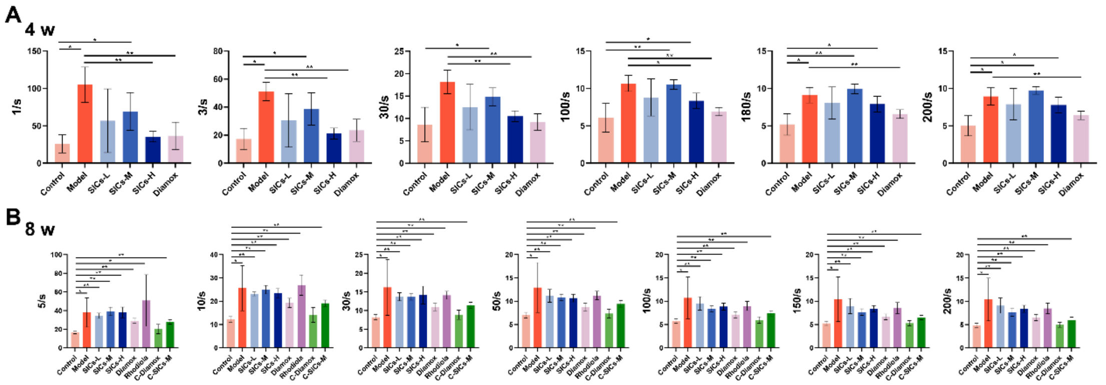

Figure S4. Changes in Blood Viscosity at Different Shear Rates in Rats Under Chronic Hypoxic Exposure (4/8 Weeks). (A) Blood viscosity at different shear rates (1, 3, 30, 100, 180, and 200/s) after 4 weeks of chronic hypoxic exposure, n = 9, * P < 0.05, ** P < 0.01. (B) Blood viscosity at different shear rates (5, 10, 30, 50, 100, 150, and 200/s) after 8 weeks of chronic hypoxic exposure, n = 9, * P < 0.05, ** P < 0.01.

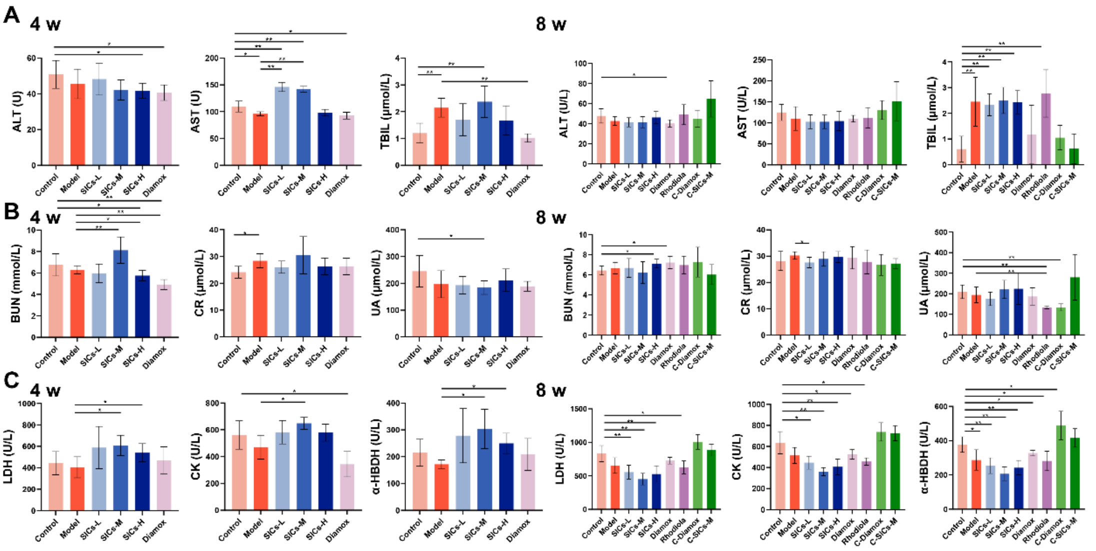

Figure S5. Selective regulatory effects of SIC on liver, kidney, and myocardial function-related indicators in rats under chronic hypoxic exposure (4 and 8 Weeks). (A) Liver function indicators (ALT, AST, and TBIL) after 4/8 weeks of chronic hypoxic exposure, n = 9, * P < 0.05, ** P < 0.01. (B) Kidney function indicators (BUN, CR, and UA) after 4/8 weeks of chronic hypoxic exposure, n = 9, * P < 0.05, ** P < 0.01. (C) Myocardial enzyme spectrum indicators (LDH, CK, and α-HBDH) after 4 and 8 weeks of chronic hypoxic exposure, n = 9, * P < 0.05, ** P < 0.01.

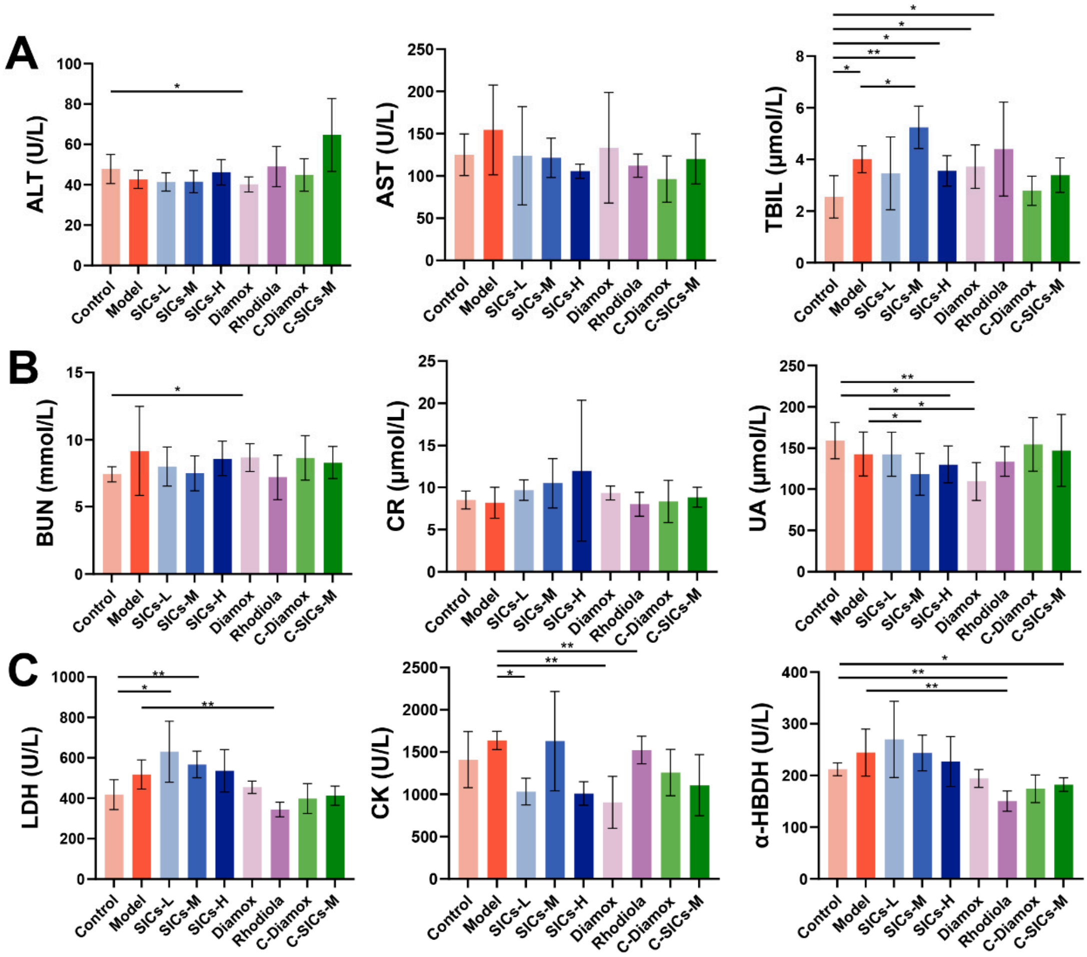

Figure S6. Effects of SIC on organ function-related indicators in mice after 8 weeks of chronic hypoxic exposure. (A) Liver function indicators (ALT, AST, and TBIL) after 8 weeks of chronic hypoxic exposure, n = 9, * P < 0.05, ** P < 0.01. (B) Kidney function indicators (BUN, CR, and UA) after 8 weeks of chronic hypoxic exposure, n = 9, * P < 0.05, ** P < 0.01. (C) Myocardial enzyme spectrum indicators (LDH, CK, and α-HBDH) after 8 weeks of chronic hypoxic exposure, n = 9, * P < 0.05, ** P < 0.01.
